# Congenital heart disease: magnitude of problem and possible interventions

**DOI:** 10.2478/abm-2021-0031

**Published:** 2021-12-30

**Authors:** Editorial Office of Asian Biomedicine

**Affiliations:** Faculty of Medicine, Chulalongkorn University, Bangkok 10330, Thailand

Congenital heart defects are among the most common congenital anomalies, representing almost 30% of all major birth defects. Congenital heart disease (CHD) has a worldwide incidence of about 9 per 1000 live births, with the highest incidence reported in Asia [1]. For 2017, the Global Burden of Disease Study reported mortality of 214,214 children with CHD aged <5 years, representing 3.97% of all causes of death and 31.5 deaths per 100,000 population [2].

Risk factors for CHD include genetic or chromosomal abnormalities, such as Down syndrome and other complex genetic factors [3]. Genetic factors are the largest known cause of congenital heart defects. Nongenetic modifiable risks include excessive alcohol consumption and use of medications during pregnancy, and maternal viral infection (e.g., rubella). Advances have been made in identifying genetic variants with possible susceptibility to environmental exposures hypothesized as an etiology for congenital heart defects [4]. Paternal characteristics and lifestyle such as maternal age, congenital heart defects in parents, smoking, alcohol consumption, maternal nutritional status, maternal obesity, maternal exposure to therapeutic drugs, parental exposure to illicit drugs, and parental exposure to environmental hazards are also implicated. Changing lifestyles have led to changes in the epidemiology of CHD [5].

Advances in cardiovascular medicine and surgery, particularly during the past 2 decades, have increased survival rates significantly and enabled many patients with CHD to reach adulthood. However, children with CHD requiring cardiopulmonary bypass surgery are at risk for neurodevelopmental impairments, which include mild impairments in cognitive and neuromotor functions, difficulties with social interaction, inattention, emotional symptoms, and impaired executive function and school performance [6]. Prenatal ultrasound screening with the involvement of specialists in fetal ultrasonography can help to improve the outcome of most CHD by providing planned delivery and perinatal management [7]. The screening can be conducted during the second trimester of pregnancy, when the mother is about 18–24 weeks pregnant.

If a child with CHD survives, measures to achieve appropriate growth, development, and improved quality of life can be achieved using standardized institutional protocols and clear guidelines to initiate feeding, promptly estimate caloric needs, and provide adequate and appropriate nutrient intake [8].

Jittham and Suwansumri [9] have described in this issue of *Asian Biomedicine* a multivariable analysis of parental risk factors associated with CHD. They found that positive family history of heart disease and maternal exposure to second-hand cigarette smoke were significantly associated with having offspring with aCHD. These findings are important to initiate family planning strategies or modify exposure to cigarette smoke for those with these risk factors. Their findings may be used to help encourage prenatal screening diagnosis, and assist planned delivery and perinatal management to promote optimal development of affected offspring.
